# Supplementary material for: Molecular mechanism of mRNA repression in trans by a ProQ‐dependent small RNA
Source: EMBO J. 2017 Mar 23;36(8):1029–45. doi: 10.15252/embj.201696127 (PMC5391140; doi:10.15252/embj.201696127)
Supplement: Supplementary file 1 — Appendix [file EMBJ-36-1029-s001.pdf]

Appendix

for

"Molecular mechanism of mRNA repression in *trans* by a ProQ-dependent small RNA"

Alexandre Smirnov, Chuan Wang, Lisa L. Drewry, Jörg Vogel

Table of contents

Appendix figures……………………………………………………………2
Appendix figure S1……………………………………………………2
Appendix figure S2……………………………………………………3
Appendix figure S3……………………………………………………3
Appendix tables…………………………………………………………4
Appendix table S1……………………………………………………4
Appendix table S2……………………………………………………6
Appendix table S3……………………………………………………8
Appendix references……………………………………………………9

APPENDIX FIGURES

Appendix Figure S1 – *In vitro* footprinting assay of the RaiZ/ProQ complex.

RaiZ is 5'-labeled. Ctr – uncleaved RNA, OH – alkaline ladder, T1 – RNase T1 ladder. Nucleotide positions are shown on the left (the first nucleotide of RaiZ-S is denoted as "1"). The decrease in the amount of the full-size RaiZ in RNase V1 reactions is likely a result of an enhanced activity of the enzyme under these conditions. The ProQ-protected sites are assigned primarily based on the Pb(II) cleavage patterns.

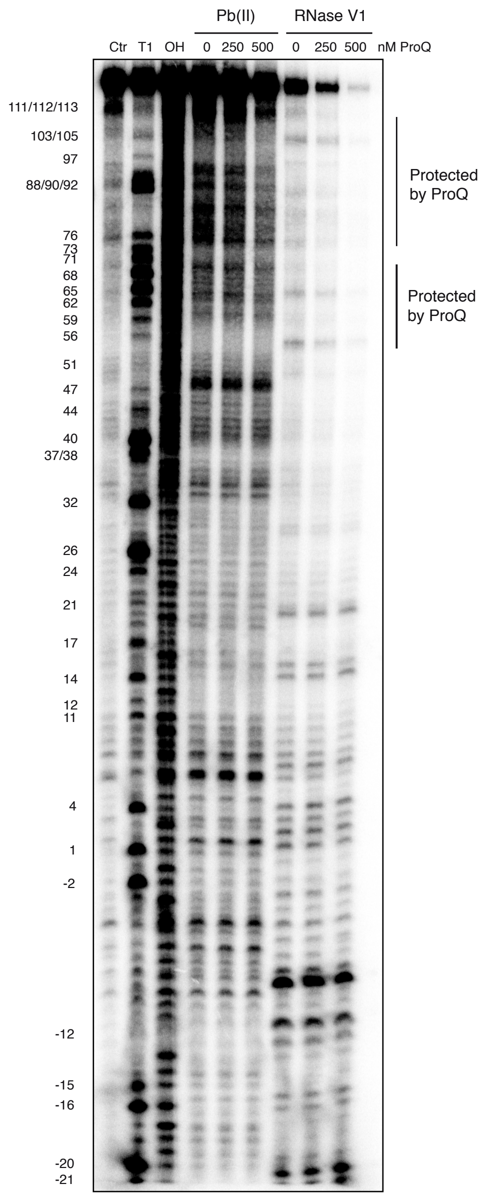

Appendix Figure S2 – Base pairing interactions between *Salmonella* RaiZ and *hupA* and *hupB* mRNAs.

Best predicted duplexes (RNAcofold; Gruber et al, 2008) between Salmonella *hupA* or *hupB* 5’ UTRs (top) and RaiZ (bottom). The start-codon is highlighted in red.

hupA - RaiZ

```
(-30)-CAAGCGAUAAACACAUUGUAAGG-AUAACUUAUG-(3)
         |||||| || || ||||||| || ||||
(101)-AACCGCUAUGUGAGUUACAUUCCCUGAUGAGAAG-(68)
```

hupB - RaiZ

```
(-114)-AUUGGGUCUGCCAC-(-107)
          ||| ||||
  (83)-AUUCCCUGAUGAGA-(70)
```

Appendix Figure S3 – Interaction between *hupA* 5’ UTR and ProQ visualised by EMSA.

Representative of three independent experiments.

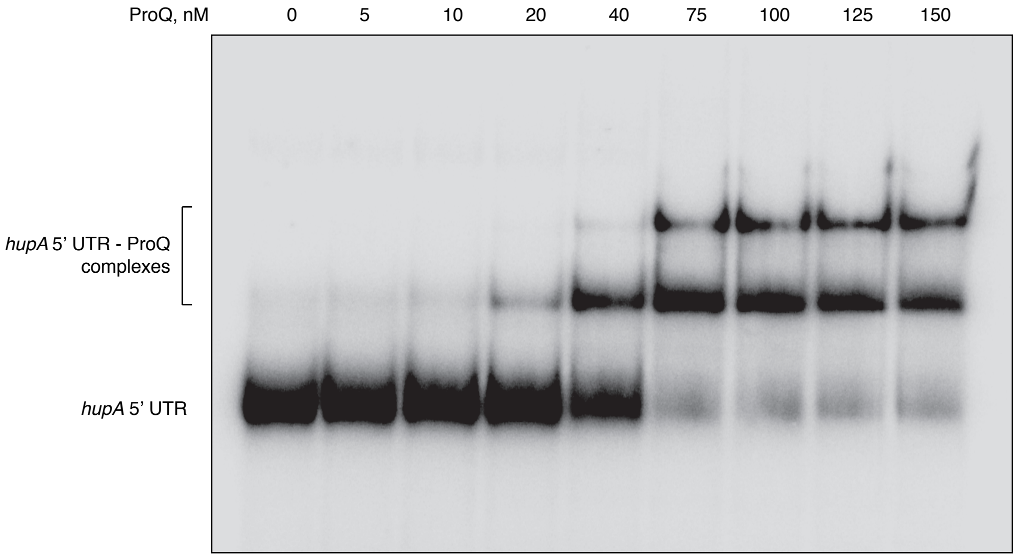

APPENDIX TABLES

Appendix Table S1. *Salmonella* strains used in this study.

Strain	Genotype	Plasmids	Description
JVS-00584	Δ*hfq*	-	*hfq* deletion strain described in (Sittka et al, 2007)
JVS-01574	WT	-	WT *Salmonella* Typhimurium SL1344 strain, parental to all other strains used in the study
JVS-03805	WT	pKP8-35	JVS-01574 transformed with pKP8-35
JVS-06999	*(rluC-rne) IG::cat*	-	WT *Salmonella* Typhimurium strain isogenic with JVS-07000 (Figueroa-Bossi et al, 2009)
JVS-07000	*(rluC-rne) IG::cat rne-3071 (ts)*	-	*Salmonella* Typhimurium strain carrying a thermosensitive *rne* allele; isogenic with JVS-06999 (Figueroa-Bossi et al, 2009)
JVS-10315	Δ*proQ::Km*^{R}	-	*proQ* deletion strain described in (Smirnov et al, 2016)
JVS-10317	Δ*proQ*	-	JVS-10315 derivative healed from the Km^R cassette; used as a principal Δ*proQ* strain; described in (Smirnov et al, 2016)
JVS-10318	Δ*hfq*Δ*proQ*	-	Strain with both the *hfq* and *proQ* genes deleted; obtained by transduction of JVS-00584 cells with JVS-10315 P22 lysates, followed by healing from the Km^R cassette, as described (Datsenko & Wanner, 2000)
JVS-10473	Δ*proQ*	pJV300	JVS-10317 transformed with the pJV300 control plasmid; described in (Smirnov et al, 2016)
JVS-10474	Δ*proQ*	pProQ	JVS-10317 complemented with the pPriQ plasmid; described in (Smirnov et al, 2016)
JVS-10582	*hupA::3×FLAG::Km*^{R}	-	WT strain with a chromosomally 3×FLAG-tagged *hupA* gene (generated with the use of oligos JVO-08601/JVO-08602 as described (Uzzau et al, 2001), validated with JVO-08603/JVO-08604 and by Western blotting)
JVS-11177	Δ*raiAZ*	-	Deletion of the entire *raiAZ* locus (generated with the use of oligos JVO-11514/JVO-11515 and healed from the Km^R cassette as described (Datsenko & Wanner, 2000), validated with JVO-11516/JVO-11517)
JVS-11178	*hupA::3×FLAG*Δ*raiAZ*	-	Δ*raiAZ* strain with a chromosomally 3×FLAG-tagged *hupA* gene; obtained by transduction of JVS-11177 cells with JVS-10582 P22 lysates
JVS-11179	*hupA::3×FLAG*Δ*raiAZ* Δ*proQ*	-	Δ*raiAZ*Δ*proQ* strain with a chromosomally 3×FLAG-tagged *hupA* gene; obtained by transduction of JVS-11178 cells with JVS-10315 P22 lysates
CWS-121	*hupA::3×FLAG*Δ*raiAZ*	pJV300	JVS-11178 transformed with pJV300
CWS-122	*hupA::3×FLAG*Δ*raiAZ*	pP_L-RaiZ-S	JVS-11178 transformed with pP_L-RaiZ-S

CWS-123	*hupA::3×FLAG*Δ*raiAZ* Δ*proQ*	pJV300	JVS-11179 transformed with pJV300
CWS-124	*hupA::3×FLAG*Δ*raiAZ* Δ*proQ*	pP_L-RaiZ-S	JVS-11179 transformed with pP_L-RaiZ-S
CWS-125	*hupA::3×FLAG*Δ*raiAZ*	pP_L-RaiZ	JVS-11178 transformed with pP_L-RaiZ
CWS-126	*hupA::3×FLAG*Δ*raiAZ* Δ*proQ*	pP_L-RaiZ^{AA}	JVS-11179 transformed with pP_L-RaiZ^{AA}
CWS-127	*hupA::3×FLAG*Δ*raiAZ* Δ*proQ*	pP_L-RaiZ^{AA}-S	JVS-11179 transformed with pP_L-RaiZ^{AA}-S
CWS-128	*ΔraiAZ*	pJV300, pXG-1	JVS-11177 transformed with pJV300 and pXG-1
CWS-129	*ΔraiAZ*	pJV300, p*hupA*-GFP	JVS-11177 transformed with pJV300 and p*hupA*-GFP
CWS-130	*ΔraiAZ*	pJV300, p*hupA*^{UU}-GFP	JVS-11177 transformed with pJV300 and p*hupA*^{UU}-GFP
CWS-131	*ΔraiAZ*	pJV300, p*hupB*-GFP	JVS-11177 transformed with pJV300 and p*hupB*-GFP
CWS-132	*ΔraiAZ*	pP_L-RaiZ, pXG-1	JVS-11177 transformed with pP_L-RaiZ and pXG-1
CWS-133	*ΔraiAZ*	pP_L-RaiZ, p*hupA*-GFP	JVS-11177 transformed with pP_L-RaiZ and p*hupA*-GFP
CWS-134	*ΔraiAZ*	pP_L-RaiZ, p*hupA*^{UU}-GFP	JVS-11177 transformed with pP_L-RaiZ and p*hupA*^{UU}-GFP
CWS-135	*ΔraiAZ*	pP_L-RaiZ, p*hupB*-GFP	JVS-11177 transformed with pP_L-RaiZ and p*hupB*-GFP
CWS-136	*ΔraiAZ*	pP_L-RaiZ^{AA}, pXG-1	JVS-11177 transformed with pP_L-RaiZ^{AA} and pXG-1
CWS-137	*ΔraiAZ*	pP_L-RaiZ^{AA}, p*hupA*-GFP	JVS-11177 transformed with pP_L-RaiZ^{AA} and p*hupA*-GFP
CWS-138	*ΔraiAZ*	pP_L-RaiZ^{AA}, p*hupA*^{UU}-GFP	JVS-11177 transformed with pP_L-RaiZ^{AA} and p*hupA*^{UU}-GFP
CWS-139	*ΔraiAZ*	pP_L-RaiZ^{AA}, p*hupB*-GFP	JVS-11177 transformed with pP_L-RaiZ^{AA} and p*hupB*-GFP
CWS-140	*ΔraiAZ*	pP_L-RaiZ-S, pXG-1	JVS-11177 transformed with pP_L-RaiZ-S and pXG-1
CWS-141	*ΔraiAZ*	pP_L-RaiZ-S, p*hupA*-GFP	JVS-11177 transformed with pP_L-RaiZ-S and p*hupA*-GFP
CWS-142	*ΔraiAZ*	pP_L-RaiZ-S, p*hupA*^{UU}-GFP	JVS-11177 transformed with pP_L-RaiZ-S and p*hupA*^{UU}-GFP
CWS-143	*ΔraiAZ*	pP_L-RaiZ-S, p*hupB*-GFP	JVS-11177 transformed with pP_L-RaiZ-S and p*hupB*-GFP
CWS-144	*ΔraiAZ*	pP_L-RaiZ^{AA}-S, pXG-1	JVS-11177 transformed with pP_L-RaiZ^{AA}-S and pXG-1
CWS-145	*ΔraiAZ*	pP_L-RaiZ^{AA}-S, p*hupA*-GFP	JVS-11177 transformed with pP_L-RaiZ^{AA}-S and p*hupA*-GFP
CWS-146	*ΔraiAZ*	pP_L-RaiZ^{AA}-S, p*hupA*^{UU}-GFP	JVS-11177 transformed with pP_L-RaiZ^{AA}-S and p*hupA*^{UU}-GFP
CWS-147	*ΔraiAZ*	pP_L-RaiZ^{AA}-S, p*hupB*-GFP	JVS-11177 transformed with pP_L-RaiZ^{AA}-S and p*hupB*-GFP
LDS-006	WT	pLD001	JVS-01574 transformed with pLD001 for arabinose-inducible RaiZ overexpression

Appendix Table S2. DNA oligonucleotides used in this study.

Name	Sequence	Description
PLlacoB	5'-CGCACTGACCGAATTCATTAA	Sense oligo to amplify pZE12-luc to generate pP_L-RaiZ/RaiZ-S (to use with PLlacoC, cover the EcoRI site)
PLlacoC	5'-[Phosphate]-GTGCTCAGTATCTTGTTATCCg	Antisense oligo to amplify pZE12-luc, to generate pP_L-RaiZ/RaiZ-S (to use with PLlacoB, begins with the (-1) position relative the promoter)
JVO-00322	5'-CTACGGCGTTTCACTTCTGAGTTC	Northern blot probe for 5S rRNA
JVO-00901	5'-TTTTTCTAGATTAAATCAGAACGCAGA	Sense oligo to amplify the pBADmyc-His A plasmid for pLD001 construction (to use with JVO-05989).
JVO-01976	5'-TCACCATCTAATTCAACAAGAATTG	Sequencing oligo complementary to a *gfp* sequence for toeprint assays.
JVO-05974	5'-GGCGATACACTCAATGTAAGGG	Northern blot probe for RaiZ (terminator region)
JVO-05989	5'-[Phosphate]-GGAGAAACAGTAGAGAGTTGC	Antisense oligo to amplify the pBADmyc-His A plasmid for pLD001 construction (to use with JVO-00901)
JVO-07333	5'-GTTTTTTATGCATGATTGGGTCTGCCACCAT	Sense oligo to amplify the *hupB* sequence (5'-UTR and first 15 codons) for cloning into pXG-10 to generate p*hupB*-GFP (to use with JVO-07334, flanked with a NsiI site)
JVO-07334	5'-GTTTTTGCTAGCATCAGCCCCTGCAGCAA	Antisense oligo to amplify the *hupB* sequence (5'-UTR and first 15 codons) for cloning into pXG-10 to generate p*hupB*-GFP (to use with JVO-07333, flanked with a NheI site)
JVO-08377	5'-CGCGCCCGAAGGCGCGTTGG	Antisense oligo to generate the RaiZ/RaiZ-S T7 transcription template (to use with JVO-10449 or JVO-08378)
JVO-08378	5'-GTTTTTTTTAATACGACTCACTATAGGAATTGATCAACAAGCTGGAAC	Sense oligo to generate the RaiZ T7 transcription template (to use with JVO-08377)
JVO-08402	5'-CACGACGGGCTTCGCC	Northern blot probe for RaiZ (5'-proximal region); used in Fig EV1
JVO-08451	5'-AATTACTGATGGCGTCATTAT	Sense oligo to validate *proQ* deletion (to use with JVO-08452)
JVO-08452	5'-GCCTGCTAATGCAAGCAGGCC	Antisense oligo to validate *proQ* deletion (to use with JVO-08451)
JVO-08564	5'-GTGTTTATCGCTTGCTAAGCATC	Northern blot probe for the *hupA* mRNA (5'-UTR)
JVO-08601	5'-GGCGTTTGTTTCTGGTAAAGCTCTGAAAGACGCAGTTAAGGACTACAAAGACCATGACGG	Sense oligo to generate a PCR product for chromosomal 3×FLAG-tagging of *hupA* (to use with JVO-08602 on pSUB11 as template), as described (Uzzau et al, 2001)

JVO-08602	5'-GGCTGATGAGCCCCTTCGATAAAACTGTTCACAGTTATGCGTCTTACATATGAATATCCTCCTTAG	Antisense oligo to generate a PCR product for chromosomal 3×FLAG-tagging of *hupA* (to use with JVO-08601 on pSUB11 as template), as described (Uzzau et al, 2001)
JVO-08603	5'-CTAACGTACCGGCGTTTGTT	Sense screening oligo to validate chromosomal 3×FLAG-tagging of *hupA* (to use with JVO-08604)
JVO-08604	5'-CAGGCAGTCAGCAACAGC	Antisense screening oligo to validate chromosomal 3×FLAG-tagging of *hupA* (to use with JVO-08603)
JVO-08605	5'-ATTGATCAACAAGCTGGAACG	Sense oligo to amplify the *RaiZ* sequence for cloning into pBADmyc HisA to generate pLD001 or into pZE12-luc to generate pP_L-RaiZ (to use with JVO-08606)
JVO-08606	5'-CACCTCTAGAAAAAAACGCGCCCGAAG	Antisense oligo to amplify the *RaiZ/RaiZ-S* sequence for cloning into pBADmyc HisA to generate pLD001 or into pZE12-luc to generate pP_L-RaiZ (to use with JVO-08605, flanked with a XbaI site)
JVO-08607	5'-CACCATGCATGCGAAAAAAAGTGGCTATCG	Sense oligo to amplify the *hupA* sequence (5'-UTR and first 25 codons) for cloning into pXG-10 to generate p*hupA*-GFP (to use with JVO-08608, flanked with a NsiI site)
JVO-08608	5'-CACCGCTAGCCAGAGCAGCTTTAGCCTGG	Antisense oligo to amplify the *hupA* sequence (5'-UTR and first 25 codons) for cloning into pXG-10 to generate p*hupA*-GFP (to use with JVO-08607, flanked with a NheI site)
JVO-10448	5'-GCAGCACAAAGGCGAAG	Sense oligo to amplify the *RaiZ –S* sequence for cloning into pZE12-luc to generate pP_L-RaiZ-S (to use with JVO-08606)
JVO-10449	5'-GTTTTTTTTAATACGACTCACTATAGGGCAGCACAAAGGCGAAG	Sense oligo to generate the RaiZ-S T7 transcription template (to use with JVO-08377)
JVO-10450	5'-GTTTTTTTTAATACGACTCACTATAGGGCGAAAAAAAGTGGCTATCGG	Sense oligo to generate the *hupA* 5'-UTR T7 transcription template for EMSAs and structure probing (to use with JVO-10451)
JVO-10451	5'-GACAGTTCTGCTTTGTCTGC	Antisense oligo to generate the *hupA* 5'-UTR T7 transcription template for EMSAs and structure probing (to use with JVO-10450)
JVO-10985	5'-GAGTAGTCCCAAACATTGAGTGTATCGC	Sense oligo to introduce the U81A,U82A double mutation in the *RaiZ* sequence (to use with JVO-10986)

JVO-10986	5'-CAATGTTTGGGACTACTCTTCTTCTGCTTC	Antisense oligo to introduce the U81A,U82A double mutation in the *RaiZ* sequence (to use with JVO-10985)
JVO-10987	5'-AACACATTGTTTGGATAACTTATGAACAAGAC	Sense oligo to introduce the A-11U,A-10A double mutation in the *hupA* 5'-UTR sequence (to use with JVO-10988)
JVO-10988	5'-AAGTTATCCAAACAATGTGTTTATCGCTTGC	Antisense oligo to introduce the A-11U,A-10A double mutation in the *hupA* 5'-UTR sequence (to use with JVO-10987)
JVO-11514	5'-ACGCATCCAGTCAAATATGGCCTGCTTTCAGCCATTCCTCTGTGTAGGCTGGAGCTGCTTC	Sense oligo to generate a PCR product for chromosomal deletion of the *raiAZ* locus (to use with JVO-11515 on pKD4 as template), as described (Datsenko & Wanner, 2000)
JVO-11515	5'-GTGTACGTAAGTACAGTACCCGTACTGTTTTAATCCTGTCCATATGAATATCCTCCTTAG	Antisense oligo to generate a PCR product for deletion of the *raiAZ* locus (to use with JVO-11514 on pKD4 as template), as described (Datsenko & Wanner, 2000)
JVO-11516	5'-CGGTATCTGATGCAACAGAA	Sense screening oligo to validate *raiAZ* deletion (to use with JVO-11517)
JVO-11517	5'-CTTGTTCGTCTTCGCATTCT	Antisense screening oligo to validate *raiAZ* deletion (to use with JVO-11516)

Appendix Table S3. Plasmids used in this study.

Plasmid	Description
p*hupA*-GFP	pXG-10 plasmid (described in (Urban & Vogel, 2007)) containing the sequence encoding the 5'-UTR and first 25 aa of *hupA* fused in frame with a *gfp* ORF under control of a P_{LtetO} promoter. The insert was amplified with JVO-08607/JVO-08608 and cloned in the NsiI/NheI sites.
p*hupA*^{UU}-GFP	Variant of p*hupA*-GFP with a A-11T,A-10T double mutation in the *hupA* 5'-UTR sequence. Generated by PCR with JVO-10987/JVO-10988 on p*hupA*-GFP as template followed by self-ligation, as described (Sharma, Papenfort et al., 2011).
p*hupB*-GFP	pXG-10 (described in (Urban & Vogel, 2007)) containing the sequence encoding the 5'-UTR and first 15 aa of *hupB* fused in frame with a *gfp* ORF under control of P_{LtetO} promoter. The insert was amplified with JVO-07333/JVO-07334 and cloned in the NsiI/NheI sites.
pJV300	pZE12-luc-derived control vector, expresses a ~50 nt nonsense transcript derived from *rrnB* terminator; described in (Sittka et al, 2007).
pKP8-35	pBADmyc HisA-derived control vector, expresses the same ~50 nt nonsense RNA as pJV300; described in (Papenfort, Pfeiffer et al., 2006).
pLD001	pBADmyc HisA-derived vector carrying a *RaiZ* sequence for overexpression under control of an arabinose-inducible promoter. The *RaiZ* insert was amplified with JVO-08605/JVO-08606 and ligated with XbaI-digested pBADmyc HisA vector amplified with JVO-00901/JVO-05989.
pP_L-RaiZ	pZE12-luc-derived plasmid expressing RaiZ under control of a P_L promoter. pZE12-luc was amplified with PlacoB/PlacoC primers, cut with XbaI and ligated with a *Salmonella RaiZ* PCR fragment, amplified with JVO-8605/JVO-08606 primers.

pP_L-RaiZ^{AA}	Variant of pP_L-RaiZ with a T81A,T82A double mutation in the *RaiZ* sequence. Generated by PCR with JVO-10985/JVO-10986 on pP_L-RaiZ as template followed by self-ligation, as described (Sharma et al., 2011).
pP_L-RaiZ-S	pZE12-luc-derived plasmid expressing RaiZ-S under control of a P_L promoter. pZE12-luc was amplified with PlacoB/PlacoC primers, cut with XbaI and ligated with a *Salmonella RaiZ-S* PCR fragment, amplified with JVO-10448/JVO-08606 primers.
pP_L-RaiZ^{AA}-S	Variant of pP_L-RaiZ-S with a T81A,T82A double mutation in the RaiZ sequence. Generated by PCR with JVO-10985/JVO-10986 on pP_L-RaiZ-S as template followed by self-ligation, as described (Sharma et al., 2011).
pProQ	pZE12-luc-derived plasmid carrying a *proQ* gene under control of its native promoter; described in (Smirnov et al, 2016).
pXG-1	Control vector for GFP-fusion reporter assay, described in (Urban & Vogel, 2007).

APPENDIX REFERENCES

Datsenko KA, Wanner BL (2000) One-step inactivation of chromosomal genes in *Escherichia coli* K-12 using PCR products. *Proc Natl Acad Sci U S A* 97: 6640-5

Figueroa-Bossi N, Valentini M, Malleret L, Fiorini F, Bossi L (2009) Caught at its own game: regulatory small RNA inactivated by an inducible transcript mimicking its target. *Genes Dev* 23: 2004-15

Gruber AR, Lorenz R, Bernhart SH, Neuböck R, Hofacker IL (2008) The Vienna RNA websuite. *Nucleic Acids Res* 36:W70-4

Papenfort K, Pfeiffer V, Mika F, Lucchini S, Hinton JC, Vogel J (2006) SigmaE-dependent small RNAs of *Salmonella* respond to membrane stress by accelerating global *omp* mRNA decay. *Mol Microbiol* 62: 1674-88

Sharma CM, Papenfort K, Pernitzsch SR, Mollenkopf HJ, Hinton JC, Vogel J (2011) Pervasive post-transcriptional control of genes involved in amino acid metabolism by the Hfq-dependent GcvB small RNA. *Mol Microbiol* 81: 1144-65

Sittka A, Pfeiffer V, Tedin K, Vogel J (2007) The RNA chaperone Hfq is essential for the virulence of *Salmonella* Typhimurium. *Mol Microbiol* 63: 193-217

Smirnov A, Förstner KU, Holmqvist E, Otto A, Günster R, Becher D, Reinhardt R, Vogel J (2016) Grad-seq guides the discovery of ProQ as a major small RNA-binding protein. *Proc Natl Acad Sci U S A* 113: 11591-11596

Urban JH, Vogel J (2007) Translational control and target recognition by *Escherichia coli* small RNAs *in vivo*. *Nucleic Acids Res* 35: 1018-37

Uzzau S, Figueroa-Bossi N, Rubino S, Bossi L (2001) Epitope tagging of chromosomal genes in *Salmonella*. *Proc Natl Acad Sci U S A* 98: 15264-9
