# Supplementary material for: Molecular mechanism of mRNA repression in trans by a ProQ‐dependent small RNA
Source: EMBO J. 2017 Mar 23;36(8):1029–45. doi: 10.15252/embj.201696127 (PMC5391140; doi:10.15252/embj.201696127)
Supplement: Supplementary file 2 — Expanded View Figures PDF [file EMBJ-36-1029-s002.pdf]

Expanded View Figures

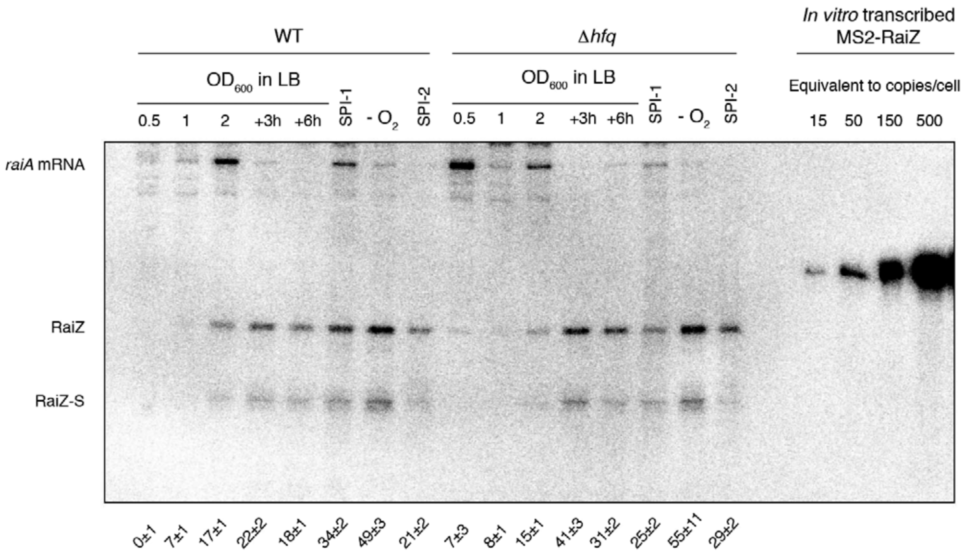

Figure EV1. RaiZ is an abundant sRNA.
Estimation of *Salmonella* RaiZ copy number throughout growth. Total RNA from ~7 × 10^8 cells was loaded in each lane along with defined amounts of *in vitro*-synthesized MS2-RaiZ (Smirnov *et al*, 2016) and probed with an oligonucleotide complementary to the *raiZ* sequence in order to evaluate the cellular abundance of *raiAZ*-encoded transcripts. Mean ± SD is shown for each condition, based on densitometric quantification.

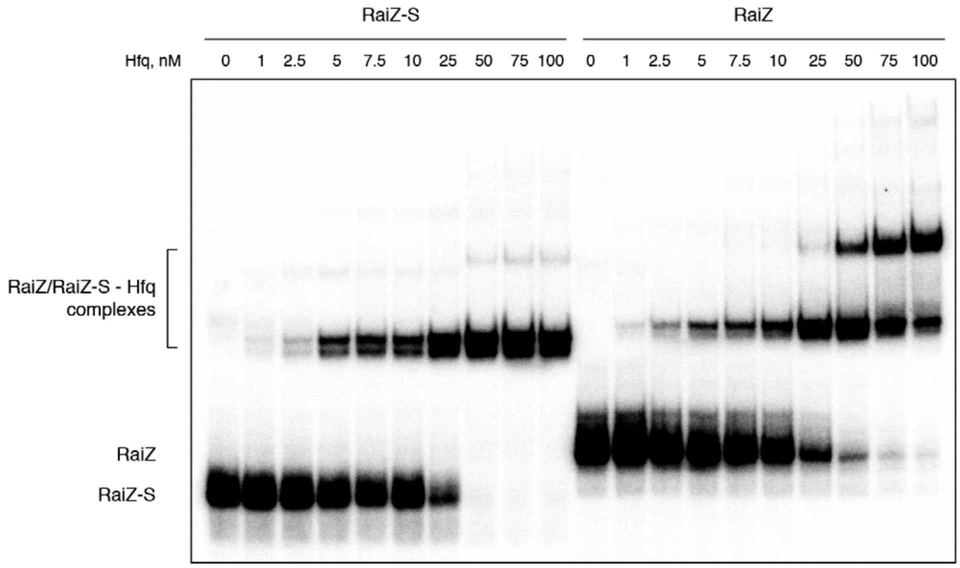

Figure EV2. Hfq forms a stable complex with RaiZ.
EMSA experiments were carried out with either the long RaiZ or the short RaiZ-S in the presence of a purified *Salmonella* Hfq protein. Apparent K_d of this complex is ~15 nM. Representative of two independent experiments.

© 2017 The Authors

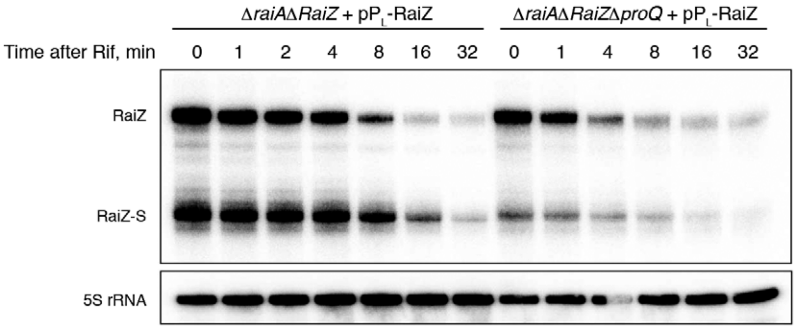

Figure EV3. RaiZ instability in a Δ*proQ* background cannot be rescued by the sRNA overexpression.
RaiZ was constitutively overexpressed in either a Δ*raiA*Δ*RaiZ* or a Δ*raiA*Δ*RaiZ*Δ*proQ* background, and its stability was assessed in the transition phase by a rifampicin assay as in Fig 3.

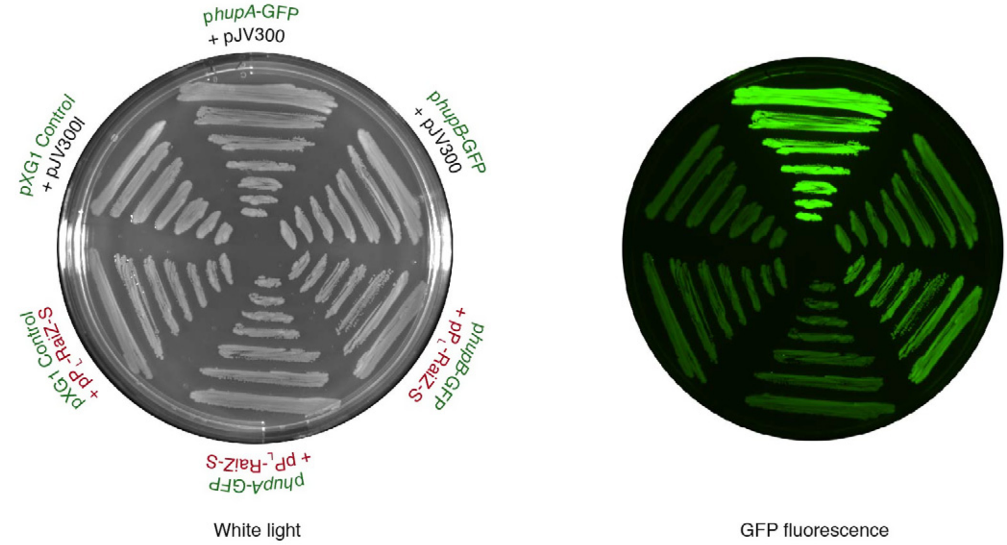

Figure EV4. RaiZ represses expression of a *hupA* fluorescent reporter construct even in the absence of Hfq.
As in Fig 4C, in a Δ*hfq* background, constitutive expression of RaiZ specifically represses a *hupA*-GFP reporter containing the *hupA* 5′ UTR and the first 15 codons of the *hupA* CDS (constitutively expressed on a pXG10 plasmid), but does not affect a *hupB*-GFP reporter.

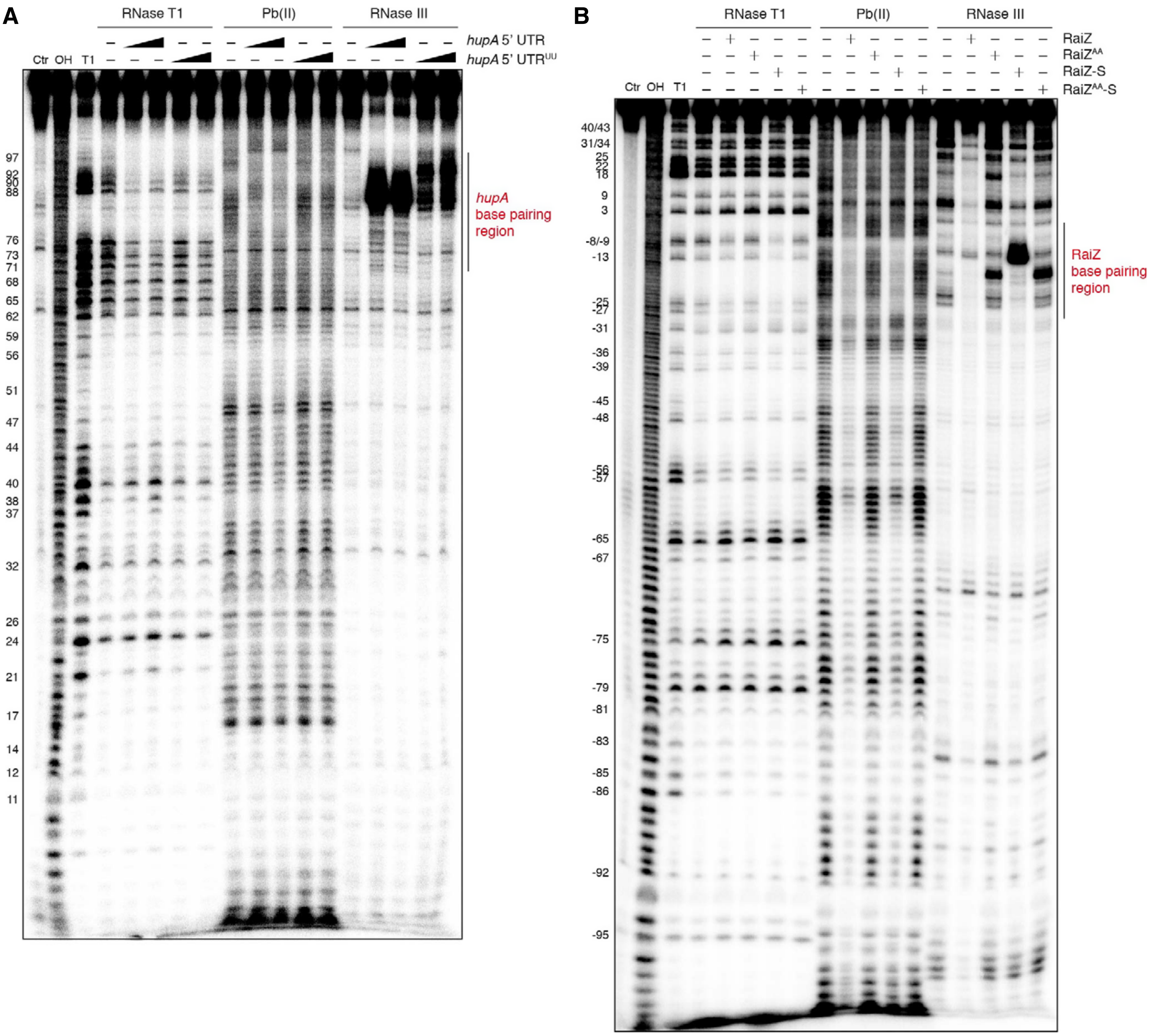

Figure EV5. Structure probing assays showing the RaiZ/RaiZ-S–*hupA* mRNA base-pairing interaction.

A Analogous to Fig 5C but with 5′-labelled RaiZ-S.

B Analogous to Fig 5C but including mutated versions of RaiZ/RaiZ-S. The *hupA* 5′ UTR and the proximal part of the CDS are 5′-labelled. Numbering on the left corresponds to Fig 5A.

© 2017 The Authors
