# Supplementary figures and images for: Molecular mechanism of mRNA repression in trans by a ProQ‐dependent small RNA
Source: EMBO J. 2017 Mar 23;36(8):1029–45. doi: 10.15252/embj.201696127 (PMC5391140; doi:10.15252/embj.201696127)

Source Data for Fig 1B

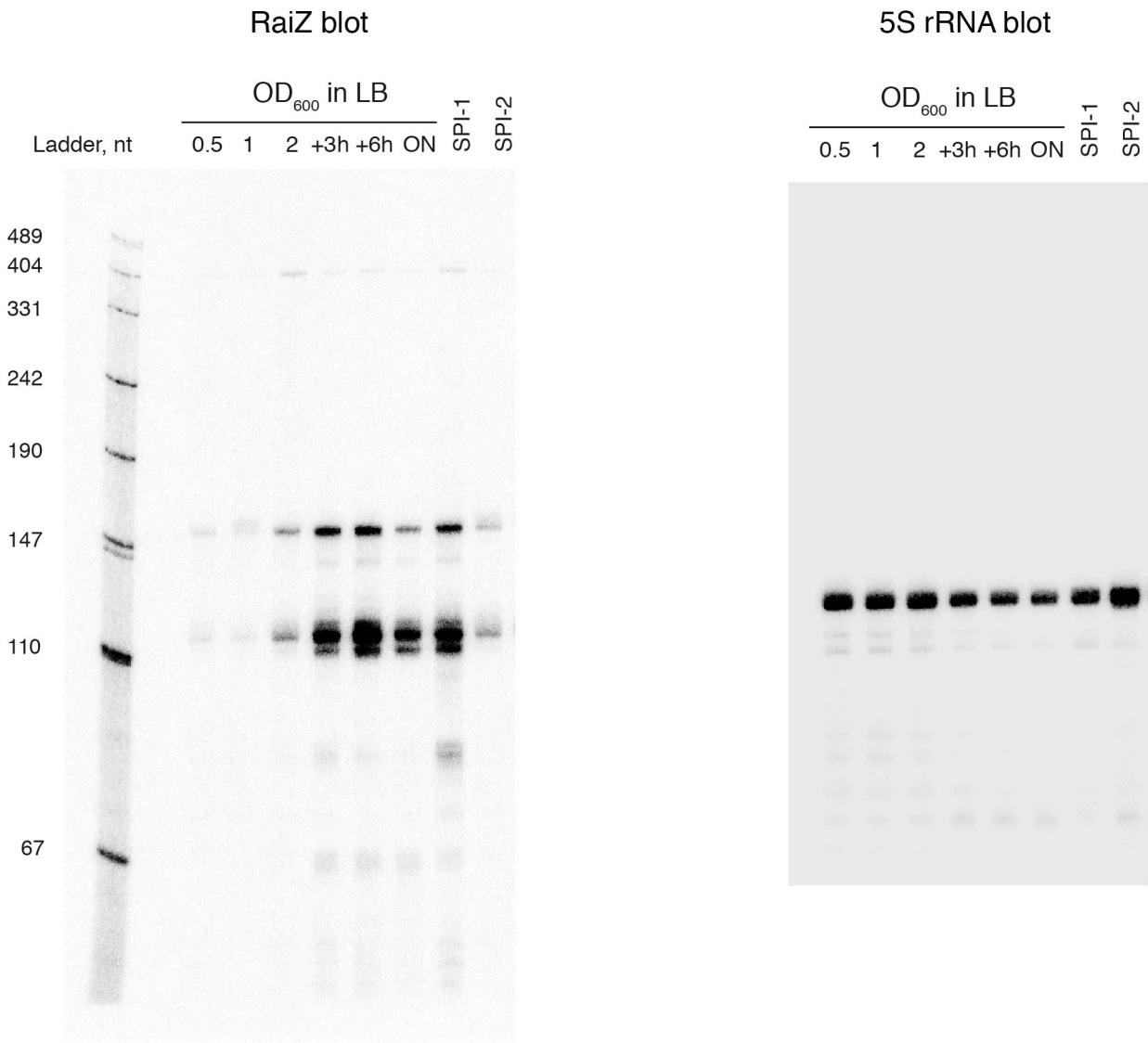

Source Data for Fig 1C

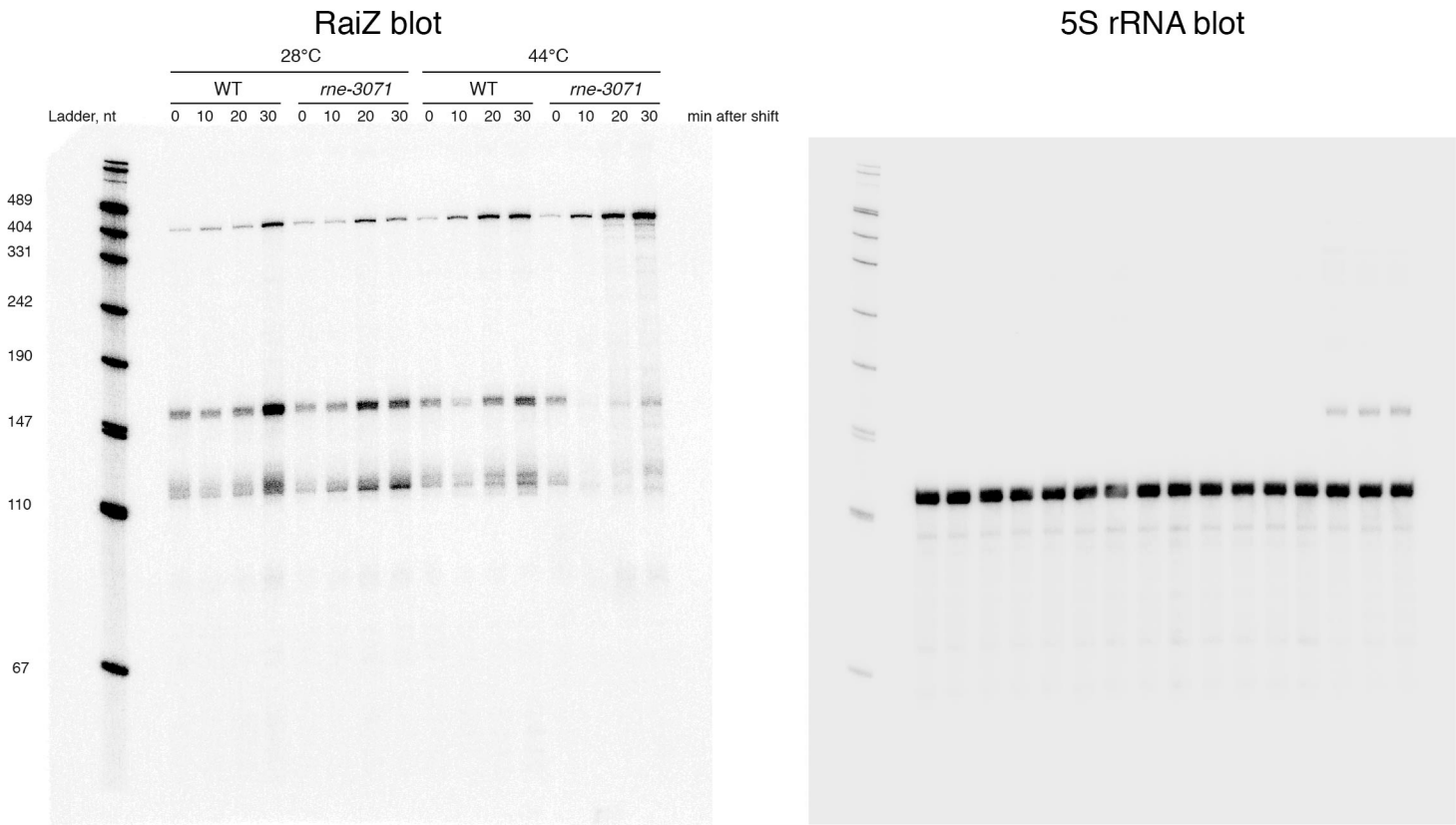

Supplement: Supplementary file 4 — Source Data for Figure 1 [file EMBJ-36-1029-s003.pdf]

Source Data for Fig 2B

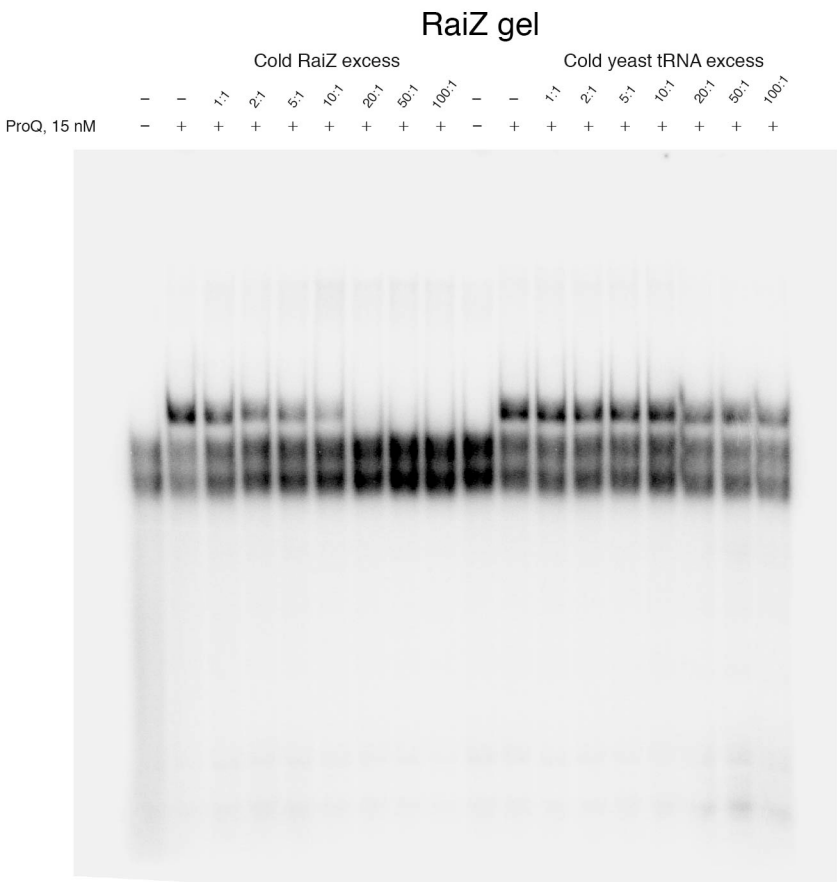

Source Data for Fig 1C

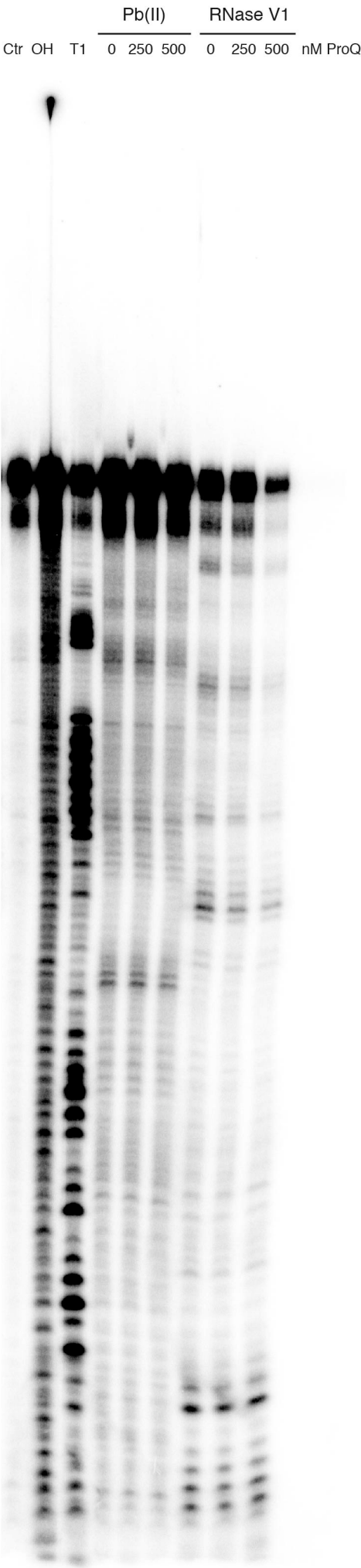

**RaiZ-S gel**

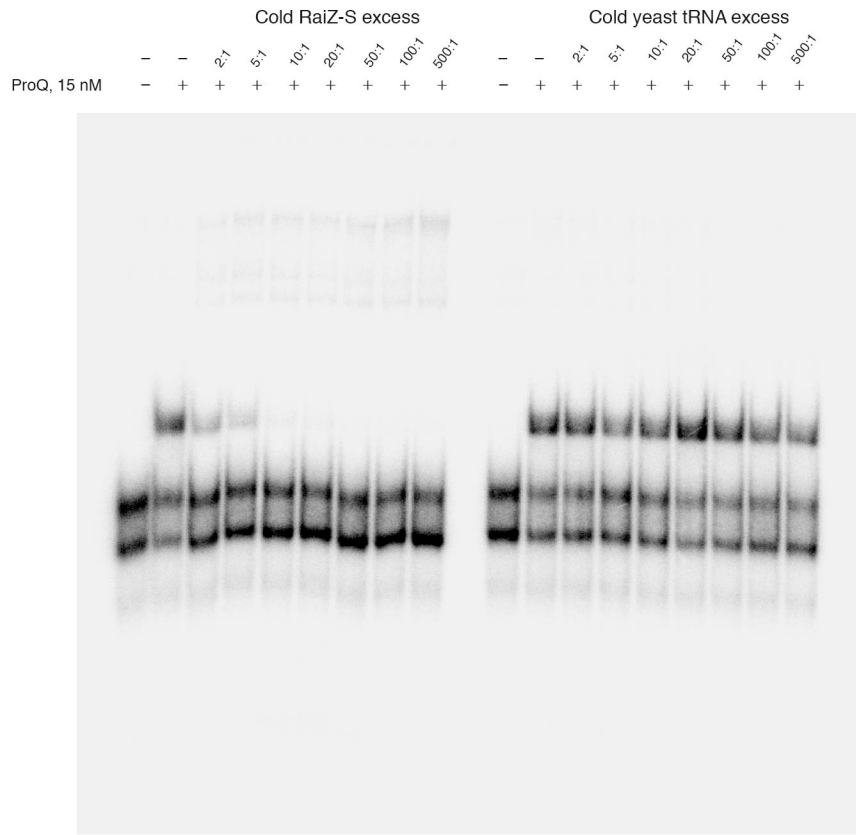

Supplement: Supplementary file 5 — Source Data for Figure 2 [file EMBJ-36-1029-s004.pdf]

Source Data for Fig 3A

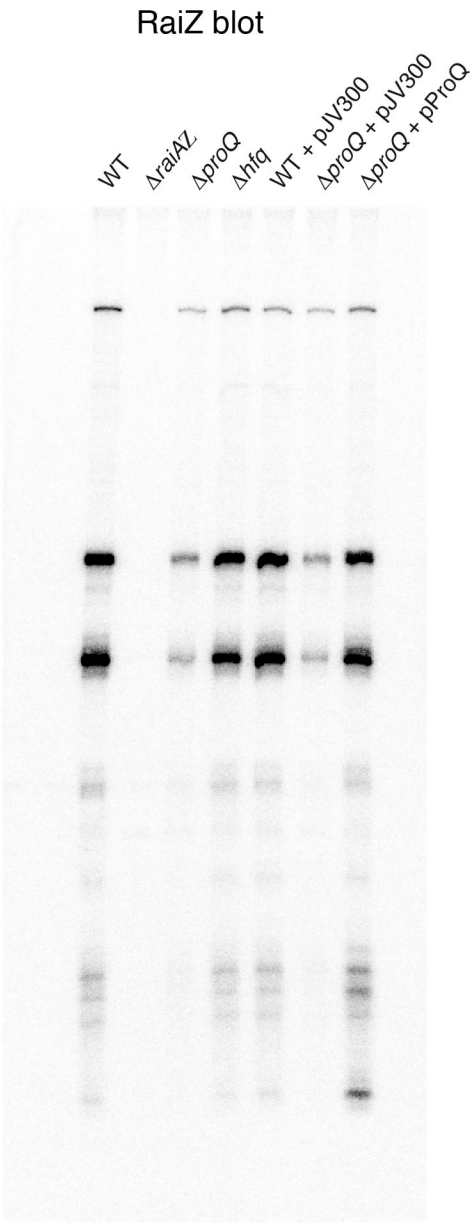

5S rRNA blot

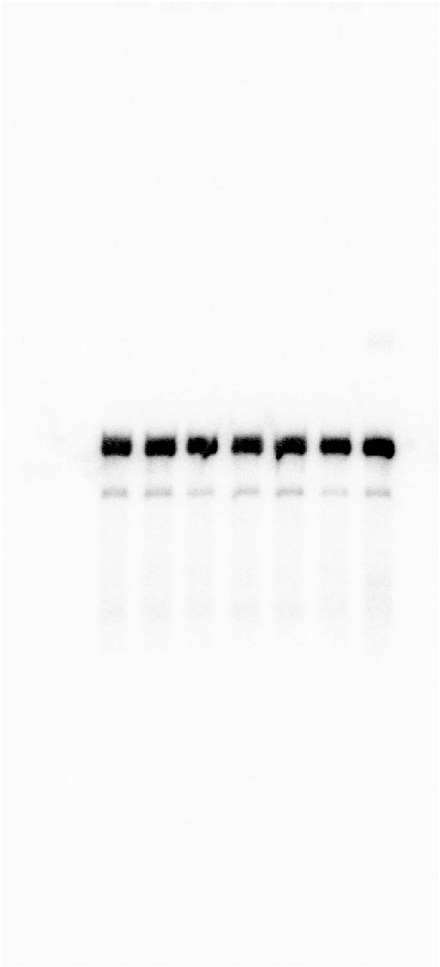

Source Data for Fig 3B

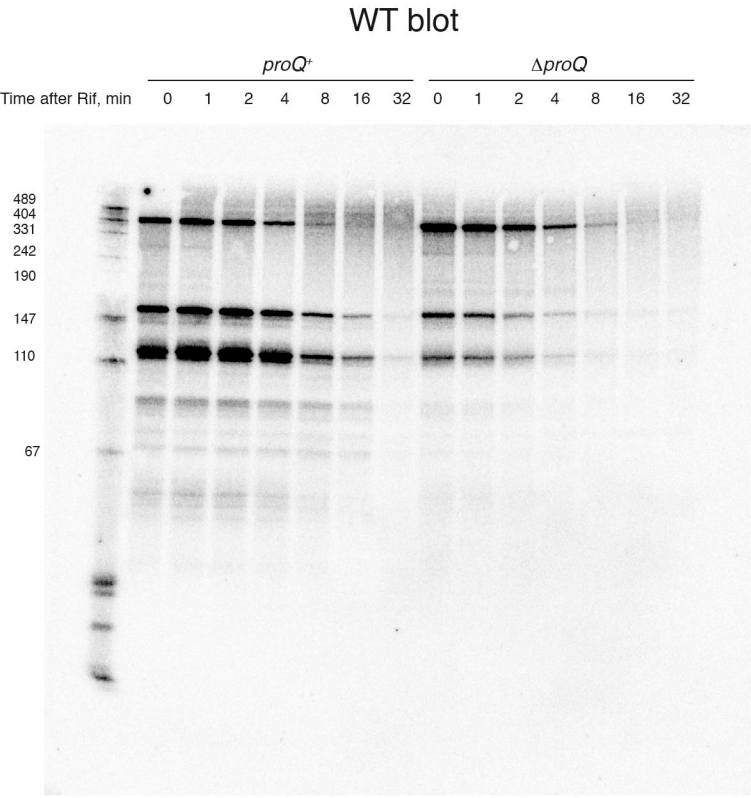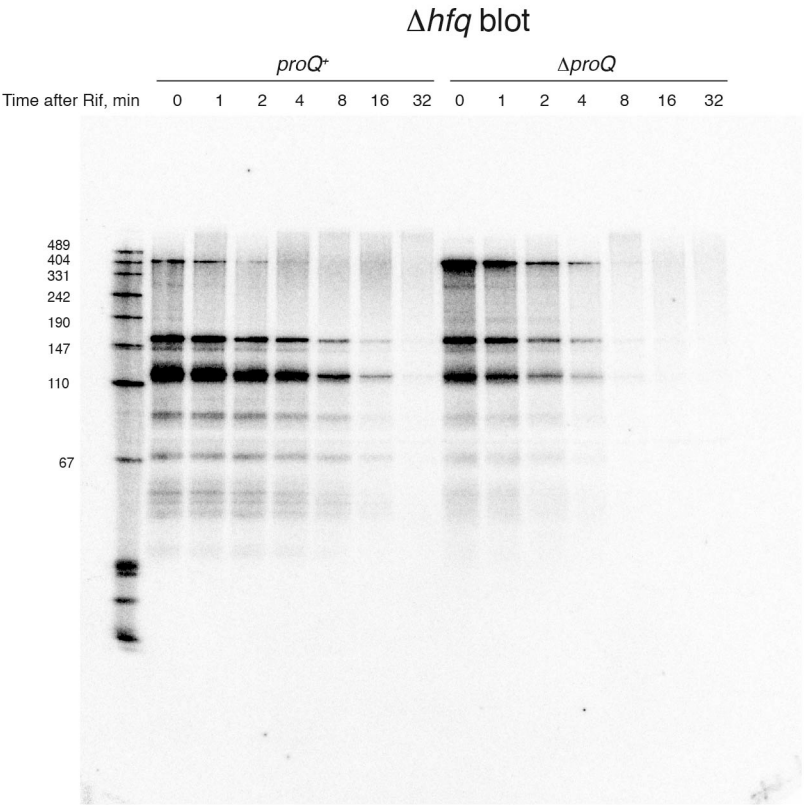

Supplement: Supplementary file 6 — Source Data for Figure 3 [file EMBJ-36-1029-s005.pdf]

Source Data for Fig 5B

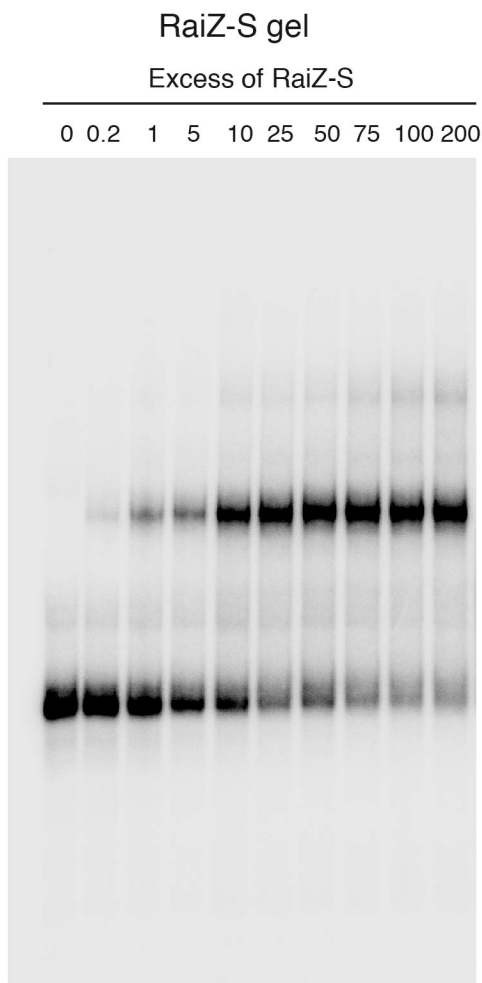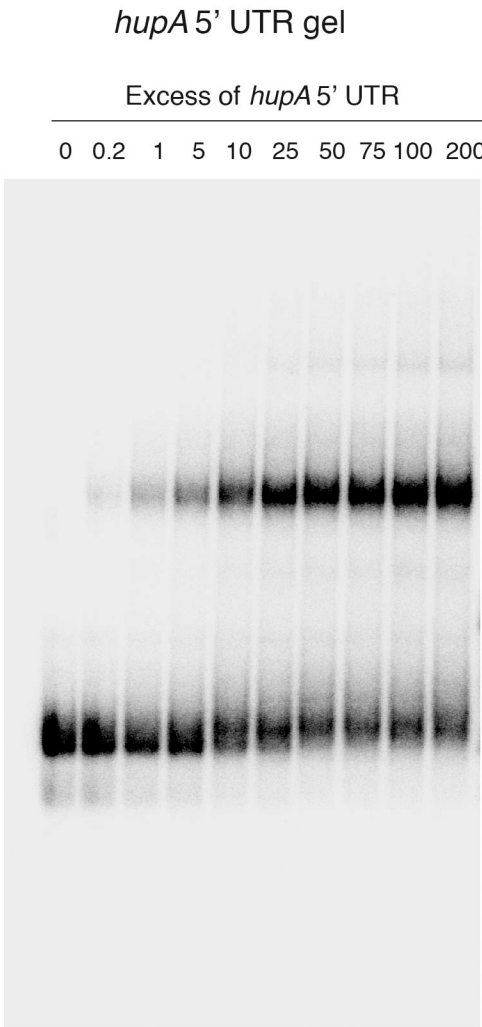

Source Data for Fig 5C

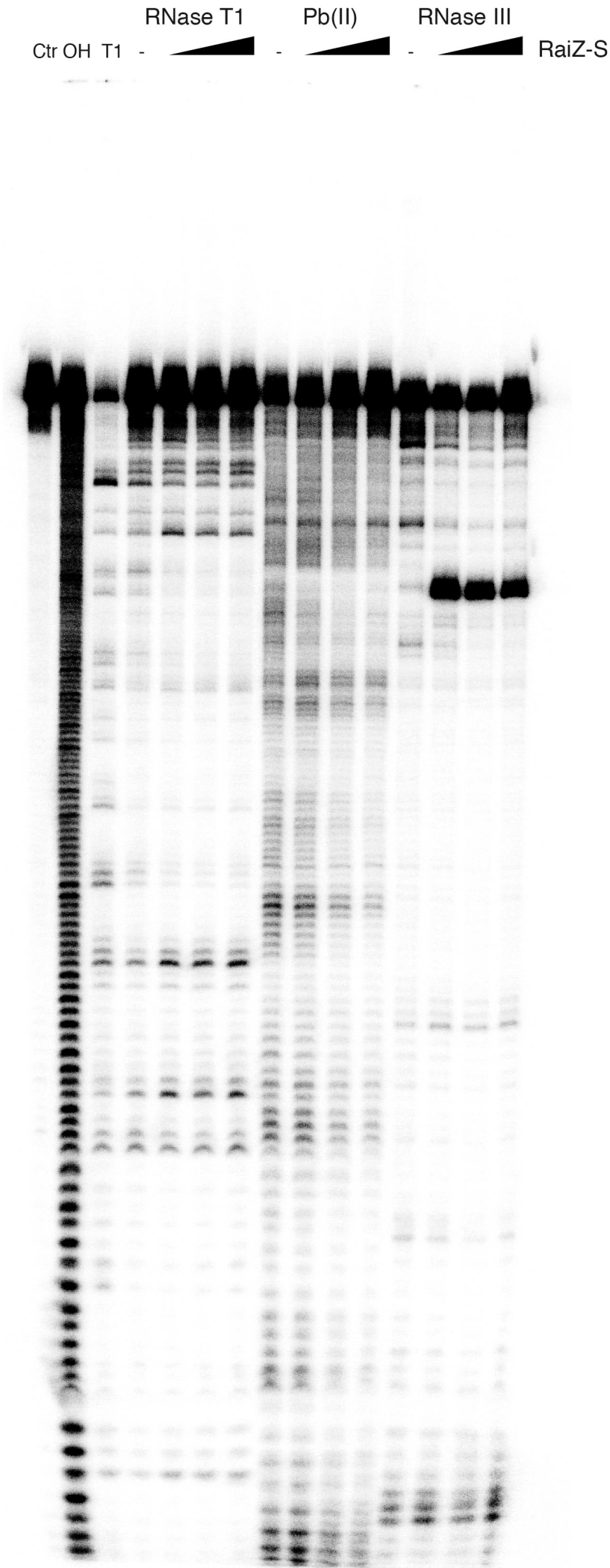

Supplement: Supplementary file 8 — Source Data for Figure 5 [file EMBJ-36-1029-s007.pdf]
