# Supplementary material for: Molecular mechanism of mRNA repression in trans by a ProQ‐dependent small RNA
Source: EMBO J. 2017 Mar 23;36(8):1029–45. doi: 10.15252/embj.201696127 (PMC5391140; doi:10.15252/embj.201696127)

Source Data for Fig 4B

HU-α-3xFLAG Western blot

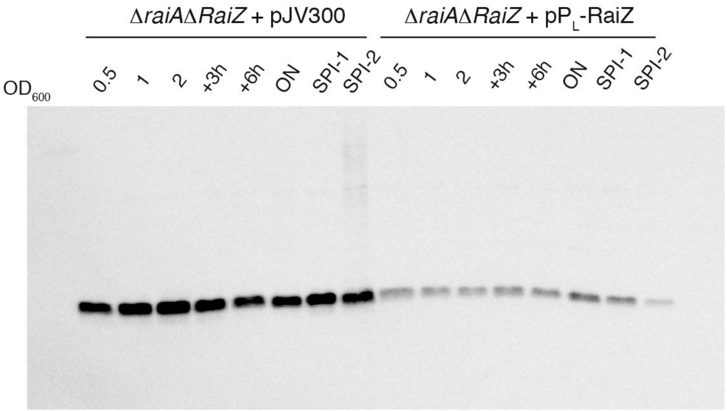

GroEL Western blot

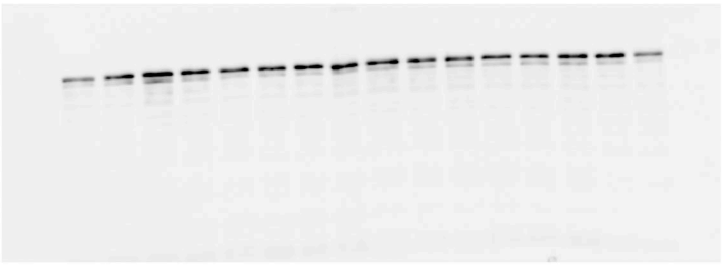

RaiZ Northern blot

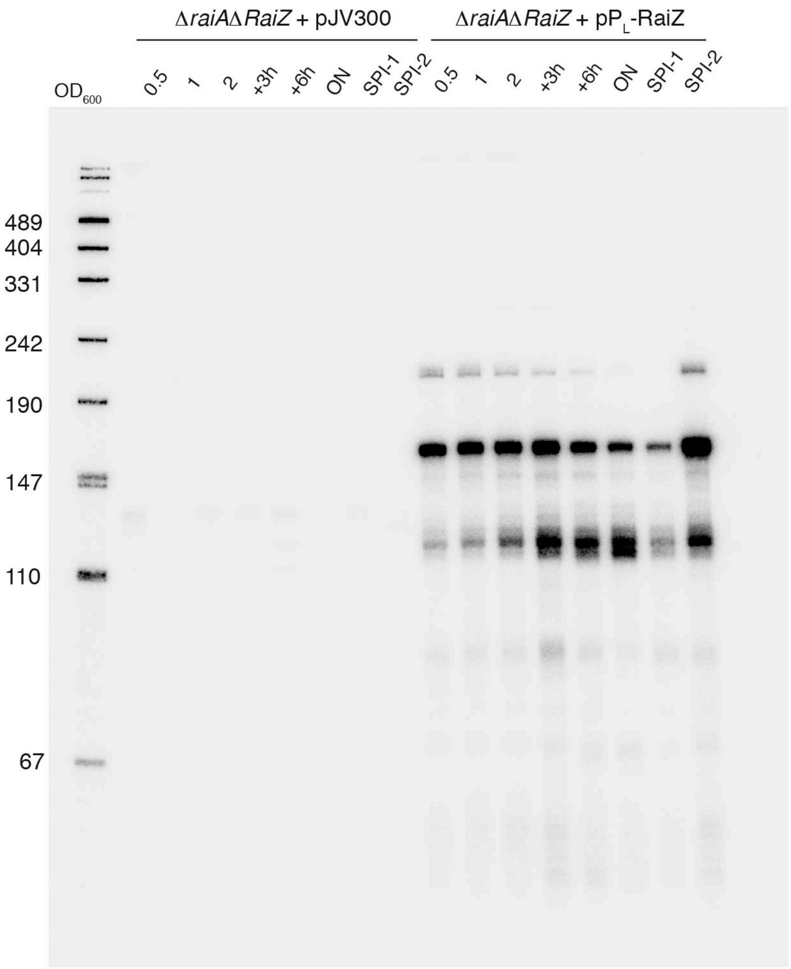

5S rRNA Northern blot

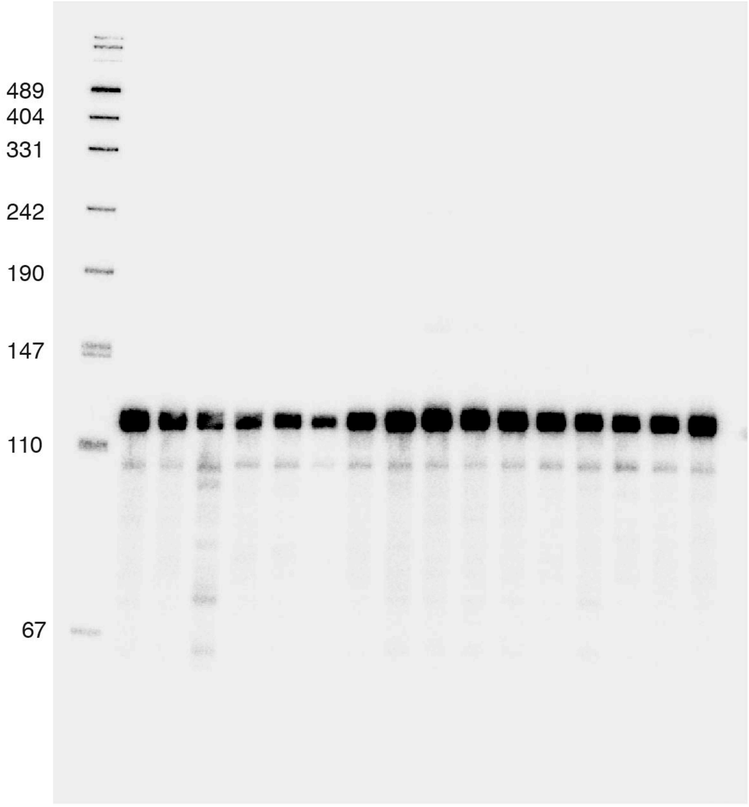

hupA mRNA Northern blot

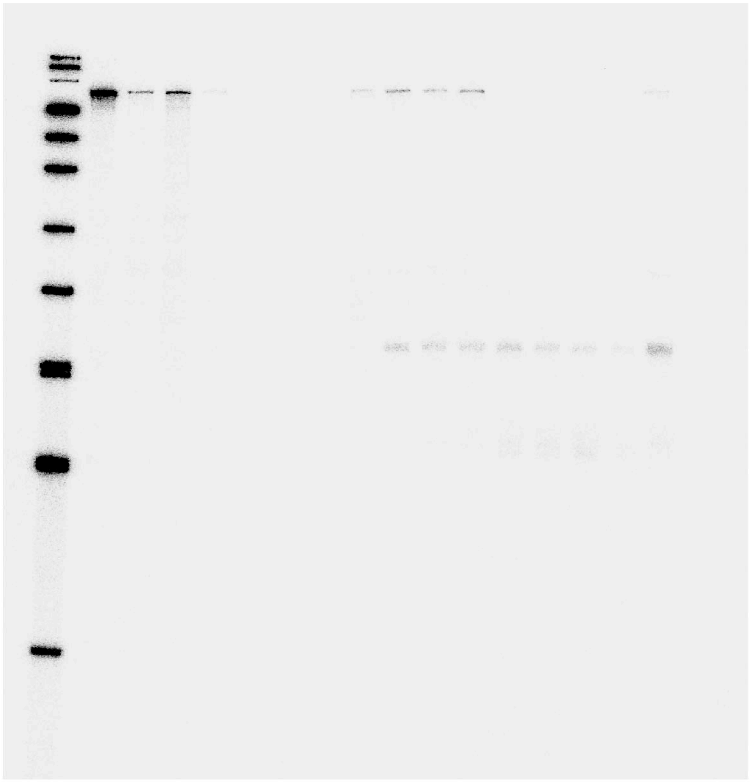

Supplement: Supplementary file 7 — Source Data for Figure 4 [file EMBJ-36-1029-s006.pdf]
